# Gastric obstruction secondary to metastatic breast cancer: a case report and literature review

**DOI:** 10.1186/1752-1947-6-232

**Published:** 2012-08-07

**Authors:** Tasadooq Hussain, Bilal Elahi, Penelope McManus, Tapan Mahapatra, Peter John Kneeshaw

**Affiliations:** 1 University of Hull; Hull York Medical School, Castle Hill Hospital, Hull, UK; 2 Breast Unit, Castle Hill Hospital, Hull and East Yorkshire NHS Trust, Castle Road, Cottingham, Hull, HU16 5JQ, UK; 3 Hull and East Yorkshire NHS Trust, Castle Road, Cottingham, Hull, HU16 5JQ, UK

## Abstract

**Introduction:**

Gastrointestinal tract soft tissues metastasis is a well-known occurrence with invasive lobular breast cancer subtypes. Gastric involvement is more common, with reports of both diffuse and localized involvements. Usually, a gastric localized involvement presents as wall thickening with an appearance similar to that of a gastrointestinal stromal tumour; rarely does a localized metastatic deposit grow aggressively to present as a large tumour causing obstructive symptoms. Our case highlights one such unusual presentation in a patient presenting with non-specific gastrointestinal symptoms. To the best of our knowledge, there have been no previous reports on a similar presentation occurring from a localized metastasis.

**Case presentation:**

A 65-year-old Caucasian woman awaiting an outpatient oral gastroduodenoscopy for symptoms of intermittent vomiting, epigastric pains and weight loss of six weeks’ duration presented acutely with symptoms of haematemesis and abdominal distension. An initial contrast-enhanced computed tomography scan showed a grossly dilated stomach with a locally advanced stenosing tumour mass at the pylorus. Our patient had a history of left mastectomy and axillary clearance followed by adjuvant endocrine therapy for an oestrogen receptor- and progesterone receptor-positive, grade 2, invasive lobular breast cancer. The oral gastroduodenoscopy confirmed the computed tomography findings; biopsies of the pyloric mass on immunohistochemistry stains were strongly positive for pancytokeratin and gross cystic disease fluid proteins, consistent with an invasive lobular breast cancer metastasis. She received a palliative gastrojejunal bypass and her adjuvant endocrine treatment was switched over to exemestane.

**Conclusion:**

Our case highlights the aggressive behaviour of a localized gastric metastasis that is unusual and unexpected. Gastrointestinal symptomatology can be non-specific and, at times, non-diagnostic on conventional mucosal biopsies. A high index of clinical suspicion in patients with a previous history of invasive lobular breast cancer can aid in an early diagnosis and treatment. A combined treatment approach with chemoendocrine therapies achieves remission and improves patient survival.

## Introduction

Distant metastasis with breast cancer to the bone, lung, brain and vulva is a known occurrence; intra-abdominal involvement is commonly seen with the intralobular histological subtype [1-3]. Clinical presentations are varied, depending on a diffuse or localized involvement. A localized presentation usually causes a segmental thickening of the involved soft tissues; by contrast, diffuse infiltrations cause bowel stricturing and present as obstructions. We report an unusual presentation of a large localized pyloric metastasis from invasive lobular breast cancer (ILC) presenting with gastric obstruction.

## Case presentation

A 65-year-old Caucasian woman with a history of ongoing intermittent vomiting, epigastric pains and weight loss of six weeks duration presented to our acute admissions unit with symptoms of haematemesis and abdominal distension. She was admitted with similar complaints a week before and was awaiting an outpatient oral gastroduodenoscopy examination. Her past history was significant for having had a left mastectomy and axillary clearance followed by adjuvant endocrine therapy for a node-positive, oestrogen receptor (ER)-positive, progesterone receptor (PR)-positive and human e growth factor receptor 2 (HER 2)-negative grade 2 ILC. On her routine follow-ups she was found to be free of any recurrence on clinical and radiological examination.

**Figure 1:**
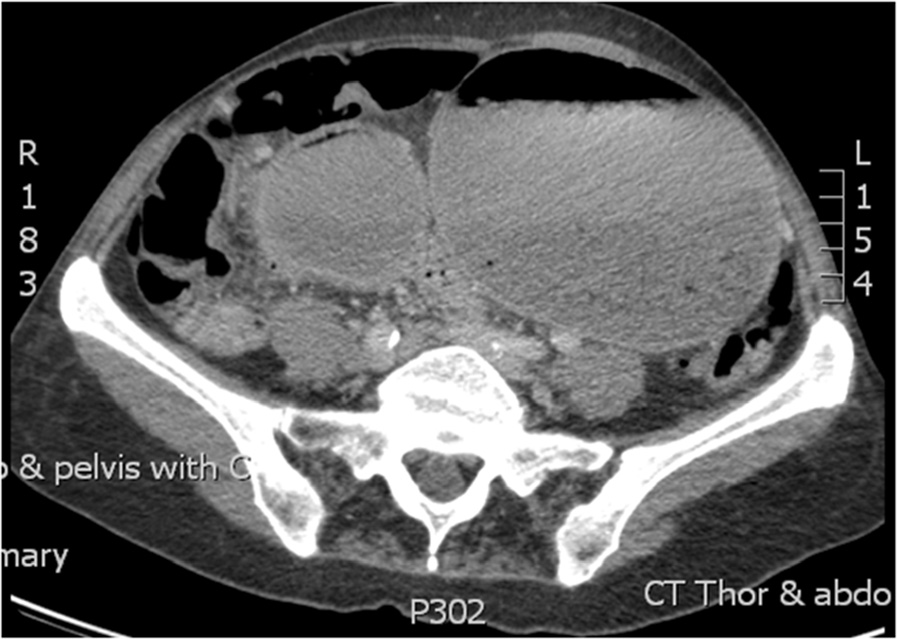
Computed tomography contrast image showing gastric outlet obstruction.

**Figure 2:**
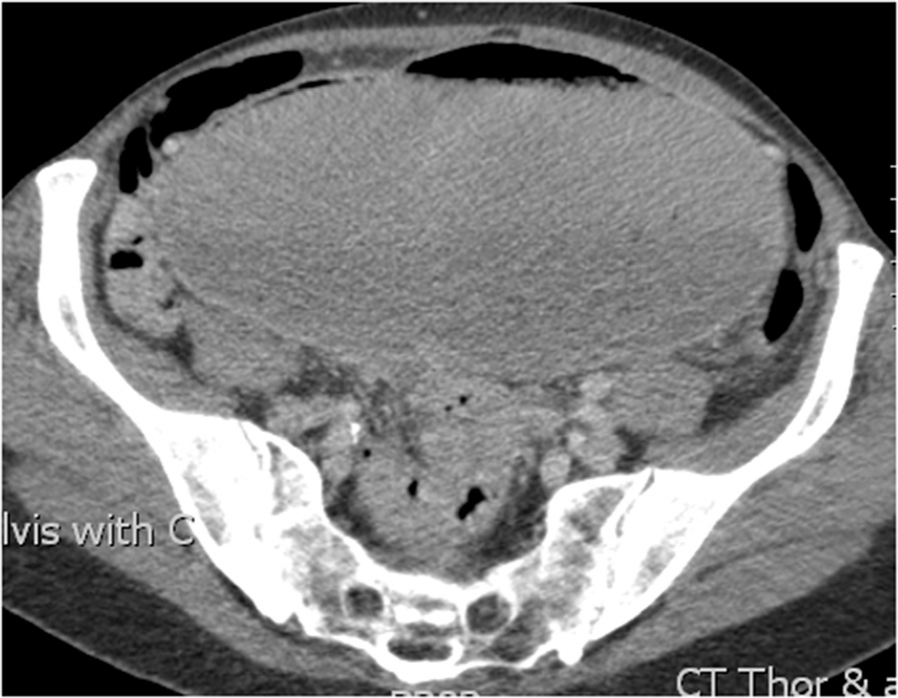
Gross distension of the stomach extending into the pelvis.

From her acute presentation, a clinical impression of a possible gastric or a high grade small bowel obstruction was made from the findings of a large gastric gas bubble on an X-ray of her chest. A contrast-enhanced computer tomography (CT) scan showed a grossly dilated stomach up to her pelvis with a locally advanced stenosing tumour mass at the pylorus (Figures 1 and 2). CT findings were confirmed on oral endoscopy with a perceived difficulty to advance the scope beyond her pylorus. Gastric biopsies from the tumour mass were taken and our patient commenced on parental nutrition. Histology from the biopsy specimen showed groups of atypical epithelial cells in small clusters, which on immunohistochemistry stain were strongly positive for pancytokeratin and gross cystic disease fluid proteins (GCDFP). A formal identification of protein receptor status (ER, PR and HER2) and Ki67 index on gastric biopsies specimens was deemed not necessary given the strong positivity to the GCDFP, a feature strongly consistent with ILC pathology. Following discussion at upper gastrointestinal (GI) and breast multi-disciplinary meetings, GI symptoms were palliated with a gastrojejunal bypass and the adjuvant endocrine treatment was switched over to exemestane.

## Discussion

ILC amongst all other invasive types is more commonly associated with GI metastasis [1]. The occurrences can involve any organ system starting from the oesophagus to the rectum [2]. The intra-abdominal organ involvements, in the order of increasing rarity include stomach, colon, rectum and small bowel. Occasionally, gastric metastasis involves peritoneum, adrenals and lung pleura presenting as primary carcinomatosis [3]. Ureteral obstructions are common presentations with retroperitoneal involvement [4]. Women affected have a median age of 55 years, with a reported mean interval of 7 years for a metastatic presentation from the time of primary diagnosis.

The initial clinical presentation can be with a primary disease or with a metastatic involvement. Usually, the breast cancer diagnosis precedes signs of metastatic progression; however, a few reports on occult breast cancer disease presenting with initial symptoms of metastatic spread have also been reported [4]. Gastric metastasis usually presents with non-specific symptoms of anorexia, vomiting, epigastric pain and - rarely - fatal haemorrhage. Endoscopy findings with diffuse gastric infiltration can mimic features of linitis plastica type infiltration, resembling a gastric malignancy; localized infiltration presents as small polyps that infrequently grow larger to resemble a gastrointestinal stromal tumour accept. Bowel involvement usually results in an obstructive pathology from the stricturing of the lumen with metastatic infiltration; occasionally, non-specific symptoms of diarrhoea and abdominal cramps precede a palpable growth [2]. Very rarely, patients can present with features of jaundice with biliary involvement from a metastatic occlusion of the distal bile ducts or duodenal infiltration [5].

The histological features of ILC are characterised by the presence of discohesive cells arranged in small clusters with a low grade nuclear pleomorphism and the absence of a stromal desmoplastic response allowing differentiation [6]. Immunohistochemistry staining for specific markers like ER, PR, cytokeratin 5 and/or 6 and GCDFP is found to be highly specific (P < 0.001, P = 0.002, P < 0.001 and P = 0.004, respectively) [6]. A further validation of the use of immunosensitivity in making an accurate pathological diagnosis comes from the immunoanalysis studies by van Velthuysen *et al*. [7]: a strong positive staining for new markers of gastric metastasis like alpha-ER, PR and negative E-cadherin (P < 0.001) help differentiate this condition from gastric adenocarcinoma.

Clinicians involved in the management of this disease should be aware of the limitations of endoscopic biopsies in excluding metastasis because of a high false negative rate from the relatively late involvement of the avascular mucosal layers with metastasis. Therefore, non-conventional techniques, like macro-biopsies or endoscopic ultrasound-guided fine needle aspiration cytology, should be employed in cases where there is a high index of clinical suspicion [3] for accurate diagnosis. The presence of non-specific GI symptomatology can usually delay the diagnosis as rarely does a localized deposit grow aggressively enough to cause obstruction of the hollow viscus. Both these features seen in our case were new and not consistent with any previously published reports. Therefore, clinicians attending to patients with a history of lobular disease should have a high index of suspicion for this diagnosis to prompt an early investigation before the patient progresses and the metastasis becomes obstructive. Medical management with systemic chemo- or endocrine therapy with or without radiation can yield better survival outcomes in young patients with non-obstructive presentations [3-7]. Surgery is usually reserved to palliate symptoms of obstruction, bleeding and perforation. However, the overall prognosis at a progressed stage from most of the reported case series is rather poor, with a predicted median survival of only 2 years from the time of diagnosis [3].

The surgical management of our patient was largely guided by her widespread intra-abdominal dissemination. Post-surgery, keeping in line with our clinical practice, the decision to switch her adjuvant treatment to exemestane was taken based on the previous ER-positive status of her breast cancer. Given our patient’s gastric biopsy specimen was strongly positivity for the GCDFP, which have a high specificity in primary lobular breast carcinoma and metastatic carcinoma of suspected breast origin [8], a routine screening for ER and PR status was deemed not necessary for the confirmation of the diagnosis or the adjuvant decision making.

Recent studies on tumour progression based on molecular characteristics have found breast tumour cells to have cancer stem cell properties regulated by HER2 protein. Studies concluded that HER2 overexpression increase the proliferation and survival of the primary tumour and have a role to play in the distant metastasis by transformation of breast tumour cells to cancer stem cells [9], thereby facilitating their migration. However, in our case, the tumour progression cannot be convincingly explained on this basis alone given the negative HER2 status of the original breast primary tumour. Both ILC and the invasive ductal cancer (IDC) subtypes have different tumour biologies explaining their metastatic patterns. Whilst IDC favour metastasising to bone, lung and liver, ILC commonly metastasises to the GI tract, lung pleura, brain and spinal cord. This behaviour, as explained by various studies, is due to the small size and shape of ILC cells, with E-cadherin overexpression favouring discohesiveness between the cells to migrate to areas of microanatomy more conducive to stopping these cells [10,11]. Alternatively, the microenvironment of the ovary or peritoneum may provide growth and survival factors that favour ILC cells over IDC cells, explaining the difference in the metastatic pattern between the two subtypes [12].

## Conclusions

Although gastric metastasis with ILC is a known occurrence, the rapid progression of a localized deposit leading to obstructive symptoms is an unusual and unexpected presentation of the disease. A high index of clinical suspicion in these patients warrants a thorough investigation of all early non-specific GI symptoms. An early diagnosis and a combined treatment approach are helpful in improving the remission and patient survival.

## Consent

Written informed consent was obtained from the patient for publication of this case report and accompanying images. A copy of the written consent is available for review by the Editor-in-Chief of this journal.

## Competing interests

The authors declare that they have no competing interests.

## Authors’ contributions

TH did the literature search and has been a major contributor in writing the manuscript. PJK did the final draft proof reading and approval. All authors read and approved the final manuscript.
